# Application of MOS Gas Sensors Coupled with Chemometrics Methods to Predict the Amount of Sugar and Carbohydrates in Potatoes

**DOI:** 10.3390/molecules27113508

**Published:** 2022-05-30

**Authors:** Ali Khorramifar, Mansour Rasekh, Hamed Karami, James A. Covington, Sayed M. Derakhshani, Jose Ramos, Marek Gancarz

**Affiliations:** 1 Department of Biosystems Engineering, University of Mohaghegh Ardabili, Ardabil 56199-11367, Iran; a.khorramifar@uma.ac.ir; 2 School of Engineering, University of Warwick, Coventry CV4 7AL, UK; j.a.covington@warwick.ac.uk; 3 Wageningen Food and Biobased Research, Bornse Weilanden 9, P.O. Box 17, 6700AA Wageningen, The Netherlands; sayed.derakhshani@wur.nl; 4 College of Computing and Engineering, Nova Southeastern University (NSU), 3301 College Avenue, Fort Lauderdale, FL 33314-7796, USA; jr1284@nova.edu; 5 Institute of Agrophysics, Polish Academy of Sciences, Doświadczalna 4, 20-290 Lublin, Poland; 6 Faculty of Production and Power Engineering, University of Agriculture in Kraków, Balicka 116B, 30-149 Krakow, Poland

**Keywords:** electronic nose, classification, chemometrics, modeling

## Abstract

Five potato varieties were studied using an electronic nose with nine MOS sensors. Parameters measured included carbohydrate content, sugar level, and the toughness of the potatoes. Routine tests were carried out while the signals for each potato were measured, simultaneously, using an electronic nose. The signals obtained indicated the concentration of various chemical components. In addition to support vector machines (SVMs that were used for the classification of the samples, chemometric methods, such as the partial least squares regression (PLSR) method, the principal component regression (PCR) method, and the multiple linear regression (MLR) method, were used to create separate regression models for sugar and carbohydrates. The predictive power of the regression models was characterized by a coefficient of determination (R^2^), a root-mean-square error of prediction (RMSEP), and offsets. PLSR was able to accurately model the relationship between the smells of different types of potatoes, sugar, and carbohydrates. The highest and lowest accuracy of models for predicting sugar and carbohydrates was related to Marfona potatoes and Sprite cultivar potatoes. In general, in all cultivars, the accuracy in predicting the amount of carbohydrates was somewhat better than the accuracy in predicting the amount of sugar. Moreover, the linear function had 100% accuracy for training and validation in the C-SVM method for classification of five potato groups. The electronic nose could be used as a fast and non-destructive method for detecting different potato varieties. Researchers in the food industry will find this method extremely useful in selecting the desired product and samples.

## 1. Introduction

The potato plant is one of the most important crops grown in the world. It is a rich source of nutrients, such as carbohydrates and protein. This product is native to South America and originated in Peru [1]. In the food industry, potatoes have a variety of applications, including baked potatoes, fried potatoes, potato crisps, potato starch, and dried fried potatoes [2].

As expectations for high-quality foods increase, it is increasingly important to determine their specifications correctly, efficiently, and purposefully. After harvesting and isolation, quality evaluation is very important in providing a reliable and uniform product to the market, as the potato plant is non-uniform in quality and maturity at harvest. Agria, Sante, Arinda, Marfona, Jelly, Bourne, Satina, Milwa, Banba, Fontane, Ramos, and Sprite varieties are the most common potato cultivars in Iran. Among these varieties, Agria cultivar potatoes have the largest cultivation area in Iran [3].

While the quality of raw potatoes is determined primarily by the size, shape, color, and desirability of the tuber, the quality of potatoes is generally determined by examining the quality of the finished product. The quality of processed products is tested by color, flavor, and texture. The quality of most processed potato products is derived from the quality of raw potatoes. Uniformity in size, shape, and composition is required for optimum quality. During storage, processing, or cooking, potatoes are exposed to a variety of phenomena affecting the product’s final quality. For consumers, the most important quality characteristics of potatoes are color, size, and texture [2]. However, quality assessment for industrial potato processing includes various parameters, such as dry matter content, starch content and its properties, and postharvest (storage) and post-processing shelf life. The variety, physical and chemical composition, and postharvest storage of potatoes are important factors that can influence the characteristics of cooked potatoes and other potato products [4].

The chemical composition and the nutritional composition of potato tubers vary with soil type, cultivars, preharvest feeding, growing seasons, storage, and adopted analysis methods [2]. Potatoes contain a large quantity of water (more than 70%), as well as starch (16–24%), protein (4%), minerals, anthocyanins, fats, and other components.

Potato has often been mentioned as a key example of a carbohydrate-rich food with various nutritional benefits. The quality of potato chips and French fries is highly dependent on different factors, such as dry matter, which is very closely related to starch content and specific weight. Moreover, sugar content can significantly determine the internal and external quality features of the fried potato products. Glucose and fructose are the main monosaccharide sugars in the potato root, with concentrations of 0.15–1.5%; they can be considered as reductive sugars [5].

Potato is usually stored at low temperatures to minimize weight loss and delay its germination. Such conditions can result in an accumulation of reductive sugar, which is a defense mechanism in response to cold weather. The increase in dissolved sugar can prevent water loss and osmotic pressure, while protecting the lipid bilayer cell membrane [6]. This response varies in different cultivars, resulting in different amounts of sugar accumulation. The acceptable limit of reductive sugar content is 1–3 g per 1 kg of fresh product. Too much reductive sugar content leads to the browning of the chips and the formation of acrylamide, a carcinogenic compound [7]. The changes in the sugar content of stored potato should be modeled at various temperatures.

At any specific time, the reduction in the sugar content of the potato is subject to the influence of various factors, such as mitochondrial respiration, the restoring of reductive sugars into starch, and starch decomposition into sucrose, which is divided into the reductive sugars glucose and fructose [5]. The reaction of starch conversion into reductive sugars includes various initial steps that can further complicate the modeling of sugar content reduction. In this regard, developed kinetic models that use time-dependent sugar data could be an appropriate alternative to the complex models used for describing the changes in sugar content reduction. In light of the complexity of the conventional methods, the development of a fast method to predict the sugar content of the various species of potato is highly essential [5]. In this regard, the electronic nose has been successfully used as a non-destructive, fast, and cheap tool for predicting the chemical parameters of various products, including carbohydrate and fiber content [8], the total soluble solid content of oranges [9], the firmness of products, the contents of sugar and acidity in the “Dabai” peach [10], the amount of volatile organic compounds (VOCs) [11], the uric acid and protein contents of wheat [12], the linalool components in products [12], and the acid and peroxide values of peanuts [13].

The olfactory machine is a system that differs in structure and approach from other methods, such as image processing and neural networking, and it is very effective in determining the quality of a variety and its classification. Moreover, olfactory systems can be used for most agricultural products, as they are characterized by odor [14,15].

Today, olfactory systems are automated, non-destructive, and cost-effective. They are ideally used for routine control and quality assurance in the food industry and for products that are related to the food industry. This technology was developed to facilitate automatic inspections using odor-based techniques; an electronic nose is a non-destructive method for measuring qualitative parameters [4].

In the present study, an electronic nose (e-nose) is used in combination with the SVM method to classify five potato cultivars. Three calibration methods—PCR, MLR, and PLSR—are also employed to predict the sugar and carbohydrate content. This approach has not been reported in any previous studies on the prediction of sugar and carbohydrate contents of different products.

## 2. Materials and Methods

### 2.1. Sample Preparation

Five potato varieties were prepared, including Agria, Sprite, Sante, Marfona, and Jelly. The data measured were the sugar and carbohydrate contents and the toughness. At the same time, the samples were evaluated with the electronic nose to determine their volatile content.

### 2.2. Electronic Nose Instrument

An electronic nose’s sensor array detects and differentiates between complex aromas of VOC emissions from biological systems. This analytical device is composed of a set of sensors, in an array, that react to volatile gases or vapors produced and released by the vegetative tissues of a biological sample. Each sensor in the sensor array responds to the VOC components that are present in a sample analyte and produces sensor outputs as electrical signals. The sensor responses are then sent to a computer system that uses multivariate data analysis methods to distinguish between the differences in the sensor data responses to the VOCs detected in the plant sample headspace, enabling unknown aromas to be classified and identified. 

**Figure 1:**
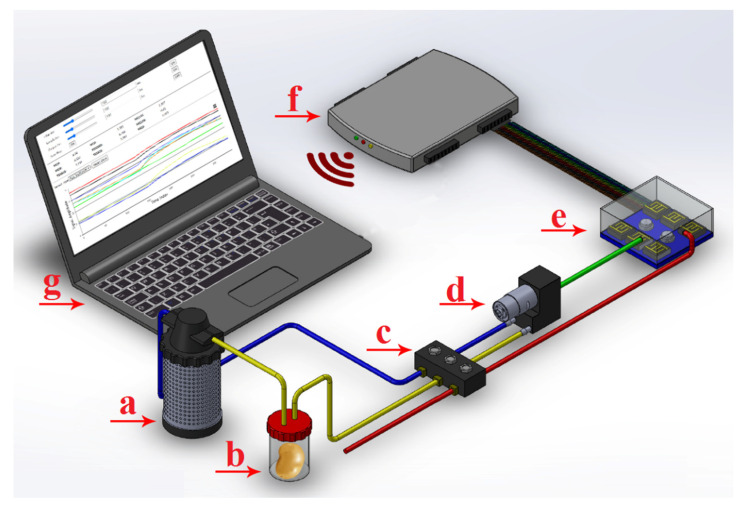
Schematic of an artificial olfactory (e-nose) system. The components of this system consist of the following parts: (**a**) air filter (activated charcoal to remove ambient-air VOC hydrocarbons), (**b**) sample headspace chamber, (**c**) solenoid air valves, (**d**) diaphragm pump, (**e**) e-nose sensor array chamber, (**f**) data acquisition recorder and wireless transmission card, and (**g**) personal computer (PC).

The electronic nose used in this study was manufactured by the Biosystems Engineering Department at Mohaghegh Ardabili University (Figure 1). The detection system consists of sensors placed inside the measurement chamber. Commercial sensors, such as metal oxide sensors (MOSs), are the most common ones available for the detection of volatile organic compounds; nine metal oxide semiconductor sensors (MOSs) have been tested [4,16]. The names of the sensors and the main applications are MQ3 (alcohol), MQ4 (urban gases and methane), MQ8 (hydrogen), MQ9 (CO and combustible gas), MQ135 (steam ammonia, benzene, and sulfide), MQ136 (sulfur dioxide), TGS813 (CH4, C3H8, and C4H10), TGS822 (steam organic solvents), and TGS2620 (alcohol and steam organic solvents). Sensors of this type have different selectivity abilities and sensitivities, but when placed in a sensor matrix, they produce a unique chemical image of the gas mixture (or “fingerprint”). The obtained data are transferred to a data acquisition system that is responsible for processing the digital signals.

#### 2.2.1. Sampling

Several potato samples of each variety (two to four potatoes) were placed in a sample container for 2 h. Each sample was tested immediately after harvest, so that the conditions for performing the tests were the same. Each potato cultivar was tested on one day. Then, the sample chamber was saturated with the odor of potatoes, and data collection was performed [17]. The data were collected by the olfactory machine in such a way that clean air was initially passed through the sensor chamber for 100 s to remove other odors. The odors of the samples were then sucked out of the specimen chamber by a pump for 100 s. then directed to the sensors. Finally, to prepare the samples for further data collection, clean air was injected into the sensor chamber for 100 s. In response to the potato odors, the output voltage of the sensors was changed and data were recorded at one-second intervals and stored in the computer. Equation (2) was used to correct the baseline to eliminate noise and possible deviations, as well as to normalize the sensors’ response [18]:

(1) $$Y_{s}(t)=\frac{X_{s}(t)-X_{s}(0)}{X_{s}(0)}$$

where *Y_S_* (*t*) is the normalized response, *X_S_* (0) is the baseline, and *X_S_ (t)* is the sensor response.

#### 2.2.2. Pattern Recognition System

The final and most important element of the electronic nose system is a pattern recognition function that assigns a series of received signals to one of the pattern classes or predicts concentrations using an appropriate mathematical calibration model. The most common models include the multiple linear regression (MLR), principal component regression (PCR), and partial least squares regression (PLSR) models. A number of explanatory and dependent variables are used to construct these models; calibration methods aim to build models that allow the quantification of traits or characteristics based on explanations of variables [19].

### 2.3. Sugar and Carbohydrate Extraction

The carbohydrate content (µg/mL) of the samples was extracted using a Nano spectrophotometer (Nanodrop) with a volume of 1000 µL and a standard glucose curve and cuvette [20]. The standard curve had a coefficient of determination of 0.9955 and the relationship was y = 0.0031x − 0.0211. For each sample, 30 replicates of data were collected and the wavelength of absorbance was used to calculate the amount of carbohydrate (by replacing the wavelength with the standard curve). In the 30 replicates, the sugar content of each sample was determined using a liquid refractometer, model BPTR100. This involved placing the potato juices on a refractometer at room temperature and measuring their sugar content in Brix. See [4] for further explanation.

### 2.4. Determination of Toughness

The toughness of the samples was determined using a proprietary tension/compression testing machine (Universal Testing Machine/STM 20, Santam Company, Tehran, Iran), which was equipped with a Bongshin load cell (model DBBP-100; Korea) with a capacity of 100 kg. First, the samples were separated from the potatoes by a cutting cylinder (15 mm long and 12 mm in diameter). The compressive tests were performed in a way that the potato samples in their most stable state were loaded quasi-elastically between two parallel plates and compressed under pre-set conditions until rupture occurred. Each potato variety was loaded at 10, 40, and 70 mm/min in seven replicates. When a sudden decrease in force occurred, as the rupture point was detected, the loading was stopped; after performing each experiment, the force-deformation curve and its related data were saved in Excel Software. Then, based on the rupture force, deformation, and specimen size, the toughness was calculated using the following equation [21]:

(2) $$KIc=\frac{E}{\pi r^{2}h}$$

where *KIc* is the toughness (MPa), *E* is the rupture energy (J), d is the deformation (mm), r is the specimen radius (mm), and *h* is the specimen height (mm).

### 2.5. Statistical Analysis

#### 2.5.1. Analysis of Variance

The obtained values for the sugar, carbohydrate, and toughness content of the five potato cultivars were analyzed using MSTATC software [Michigan State University, USA]. The statistical analyses were conducted using a completely randomized factorial test. The means were compared using Duncan’s multiple range test at an 0.01 *p*-value level.

#### 2.5.2. Chemometric Analysis

Chemometrics uses multivariate statistics to extract useful information from complex analytical data. To model and explain the relationship between odor data and chemical properties (sugar, carbohydrate, and toughness), multivariate calibration models using the PLSR, MLR, and PCR methods were used in this study. A one-variable calibration includes only one dependent variable and one independent variable. In this study, x was considered as the independent variable (output data from the sensors) and y was considered as the dependent variable (samples measured in the laboratory, i.e., percentage of sugar, carbohydrate, and toughness). The simplest condition is determined as follows:

(3) $$y=b_{0}+a_{1}.x_{1}+a_{2}.x_{2}+\ldots +a_{n}.x_{n}+e$$

The coefficients “*a* and *b*” can be calculated using the partial least squares regression method. In this model, “*b_0_*” is the intercept and the coefficient, “*a*” is the beta coefficient, and “*e*” is the calibration error due to the measuring of the response error. This model is called the classical model. The one-variable method is used when the sample is simple and free from any disturbance. The calibration methods include the following univariate methods: partial least square regression (PLSR), multiple linear regression (MLR), and principal component regression (PCR).

The MLR model establishes a linear relationship between a dependent variable (y) and a set of multiple explanatory variables (x). This model can be used when the number of variables is less than the number of samples and there is a poor relationship between them. Otherwise, it is impossible to estimate the regression coefficient “*a*” using the least-squares method [19]. The MLR model is used to calculate the sensor regression coefficients to minimize the total squared deviations (gradient descent) between the predicted quality indicators and the actual quality indicators [21].

The PCR model reduces the number of explanatory variables by selecting several principal components (PCs) instead of the main variables. The main idea of this method is to establish the relationship between the PCs and the expected characteristic of the sample. It is possible to apply this method in two stages. The first stage involves identifying the principal components using principal component analysis (PCA). Thus, we obtain unrelated matrices of variables. In the second

Partial least squares regression (PLSR), also known as PLS, is a new type of multivariate statistical analysis method. It is the most common method for developing multidimensional calibration models. It can process linear data and reduce the number of calibration samples required by the gold standard in chemistry [22]. When the dependent variables have a higher linear correlation, PLS can be more effective. PLS is a two-line model based on the matrix x (independent variables) and Y (dependent variables), which can be considered as external and internal relations [21].

A support vector machine (SVM) is a supervised learning model with a classification algorithm based on statistical theory. An SVM can nonlinearly plot data that cannot be linearly separated in a small space into a large space, using a kernel function. A hyperplane is created in a high dimensional space to maximize the distance between two classes and classify data in the high dimensional space. Radial basis functions (RBFs) are the most common kernel functions that lead to a high classification function. Since an SVM minimizes structural risks, it is considered a good classifier for nonlinear data and small samples [14,23].

In this study, the results from nine sensors were used as independent variables x, with sugar, carbohydrates, and toughness as dependent variables y. Unscrambler *X* 10.4 software (Unscrambler version *X* 10.4, CAMO, Trondheim, Norway) was used. First, PCR and PLSR analyses were performed; then, after determining the optimal parameters, PCR and PLSR models were written using the MLR model, considering the optimal parameters as independent variables.

### 2.6. Model Performance

To quantify the predictive power of the models, the coefficient of determination (R^2^) and the root-mean-square error (RMSE) were calculated. In this experiment, the performance of the models was evaluated using the cross-validation technique. This technique is usually used when a data set is small and limited. In using this technique, a single observation is selected from all the items as a validation item, and the predictive performance is extracted from the other data. This process is repeated for all the observations, so that all the samples are used once as a validation item [24]. Thus, the model is calibrated with as many samples as possible. In addition, all data are used for model calibration and validation.

## 3. Results and Discussion

### 3.1. Sugar and Carbohydrate Content of Potato Varieties

The sugar and carbohydrate content and the toughness of five different potato varieties were measured using a refractometer, the Schiegel method [4], and an STM device. The related data were analyzed using Mstatc software. The results are presented in Table 1:

ANOVA results for the sugar and carbohydrate content and toughness of the five different potato varieties was significant at 0.01, with the following coefficients of variation: 0.27%, 7.67%, and 2.28%, respectively.

Table 2 illustrates the average Brix sugar (gram of sugar per 100 g of solution), carbohydrates (μg/mL), and toughness (MPa) of the potato varieties. According to Table 2, the Sprite variety had the highest sugar content (8.15 Brix) and the Agria and Jeli varieties recorded the lowest sugar content, with 6.18 and 6.12 Brix, respectively. The highest carbohydrate contents were observed in the Sprite, Sante, and Jelly varieties, with 276.7, 266, and 237 μg/mL, respectively, while the lowest values were recorded in the Marfona and Agria varieties. At a loading rate of 10 mm/min, the Marafona variety showed the highest value for toughness. According to the results, the Agria and Sprite varieties showed lower toughness. This may be explained by the fact that the higher the carbohydrate content in a variety, the crispier and less firm the variety.

The sugar content of different varieties varies because starch (the main ingredient in potatoes) is hydrolyzed differently as the plants respire. The lower the starch content of a variety, the less sugar it contains. In addition, the chemical composition varies depending on the potato variety, the soil, the climate, and agronomic factors. Generally, it is believed that potatoes with more sugar are suitable for the French fry industry, while potatoes with medium sugar content are suitable for deep frying [24]. The highest amount of carbohydrates was observed in three potato cultivars, Jelly, Sante, and Sprite, and the Sprite cultivar had the highest amount of sugar. The difference in sugar content of different types of potatoes is due to the difference in the hydrolysis of starch, which is caused by the respiration of the product. The lower the starch content in a cultivar, the lower the sugar.

Gumul, et al. [25], measured the sugar content of five different potato varieties. They found that the lower the sugar content of the different potato varieties, the lower the quality of the product, because at high temperatures the sugar reacts with maillard, leading to the formation of potential substances that are hazardous to human health.

### 3.2. Response Sensors

**Figure 2:**
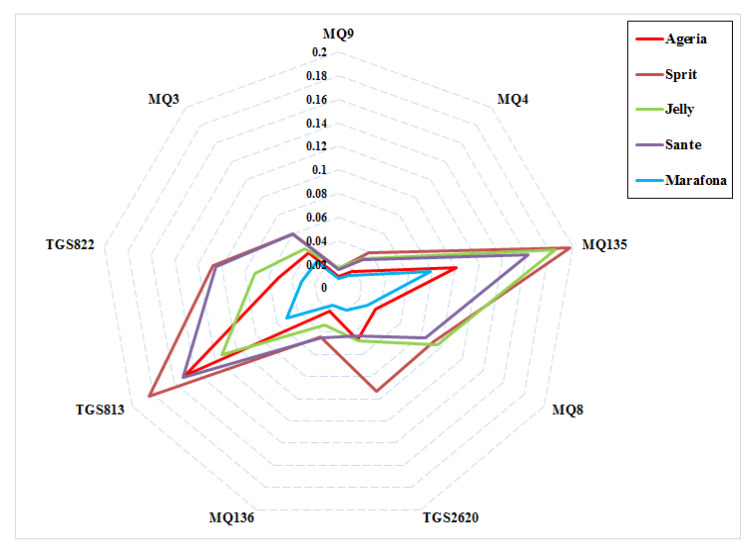
Radar raw fingerprint chart (sensor intensities) of the VOCs potato cultivars.

Figure 2 demonstrates the output response of the sensors to the odor of the potato samples. Radar diagrams were used to observe the differences in patterns (fingerprints) between different potato cultivars. The average output data of electronic nose sensors, during 100 s of measurements, were plotted as a radar diagram following normalization using Equation (1). Using this diagram, it is possible to visualize the difference between the response patterns of the sensors to the odor of each potato cultivar. According to the radar graph, there is a little similarity in the fingerprints of different potato cultivars. Accordingly, the highest odor is related to the Sprite, Sante, and Jelly cultivars. These three cultivars also had the highest carbohydrate content. It is likely that the reason for the greater odor of these three cultivars is their higher carbohydrate content. The gas sensors MOS, TGS813, and MQ135 showed the highest response to sample odor, while the sensors MQ136, MQ4, and MQ9 showed the lowest response.

The gas sensors, MOS, TGS813, and MQ135, showed the highest response to sample odor, while the sensors MQ136, MQ4, and MQ9 showed the lowest response. 

### 3.3. Prediction of Quality Parameters of Carbohydrate and Sugar Based on PLSR and PCR

The relationship between electronic nose signals and the prediction of sugar and carbohydrate content was described by the PLSR and PCR models. The performance of the PLSR and PCR models for classification prediction was evaluated using R2 and RMSE. The root-mean-square error validation (RMSEval) of the root was chosen as a numerical tool to select the optimal model (Table 3). The highest accuracy of prediction of the sugar and carbohydrate parameters was related to the Marfona cultivar. Both models, PCR and PLSR, were able to predict the amounts of sugar and carbohydrates with an accuracy of about 0.90, with only one optimal factor. Both models were very accurate in predicting the amount of carbohydrates and sugars.

### 3.4. Prediction of Quality Parameters of Carbohydrate, Sugar and Toughness Based on MLR

The multiple linear regression (MLR) method lies between the PLSR and PCR methods in terms of mean squared error; it was used to build the PLSR and PCR models. After analyzing and determining the optimal factors, the PCR and PLSR models were considered as independent variables and the MLR model was used. The MLR model described the relationship between the sensor signals and the carbohydrate and sugar parameters. The remaining variables were all significant at the 0.01 level. Then, different statistical methods were used to test the final equations for stability and validity. Three criteria were used to select the correct equation for further analysis: R^2^, RMSE, and the number of descriptors in the model. The leverage correction method, which uses cross-validation, was used instead of the PLSR and PCR validation methods.

**Figure 3:**
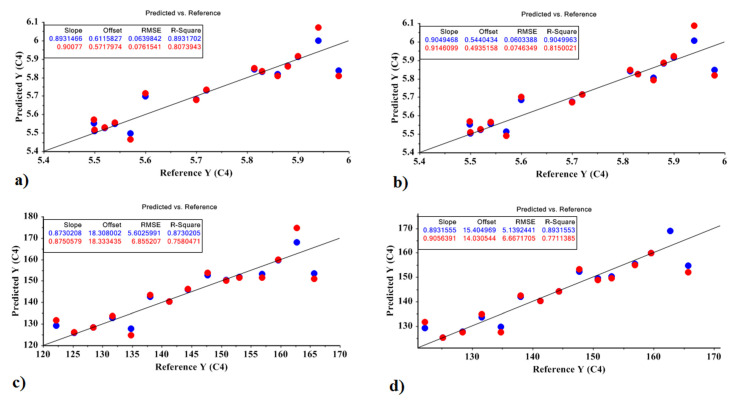
MLR prediction models for predicting the amount of carbohydrate and sugar in the Sprit cultivar: (**a**) the amount of sugar based on the PCR method; (**b**) the amount of sugar based on the PLSR method; (**c**) the amount of carbohydrate based on the PCR method; (**d**) the amount of carbohydrate based on the PLSR method.

For the best MLR models, the standard error of prediction is low, the F and R^2^ values are high, the predictive power is high, and the descriptors are minimal. Correlation plots are shown in Figure 3 as a visual method to evaluate the models’ fit with experimental data. Equations (4)–(7) represent the model obtained by the MLR model based on the optimal factors of the PCR model and the PLSR model for predicting the amount of sugar and carbohydrate in the Sprit cultivar:

Sugar based on PCR = 5.723 − 9.679 X1 − 10.202 X3 + 0.005(4)

X2 was not significant at the 0.01 level, so X2 were deleted in the model. The values obtained for the model were R^2^ = 0.807 and RMSE = 0.076; *p* < 10^−4^.

Sugar based on PLSR = 5.723 − 9.865 X1 + 9.846 X2 + 0.005(5)

X3 was not significant at the 0.01 level. The values obtained for the model were R^2^ = 0.815 and RMSE = 0.074; *p* < 10^−4^.

Carbohydrate based on PCR = 144.181 − 740.697 X1 − 991.713 X3 + 46.993(6)

X2 was not significant at the 0.01 level. The values obtained for the model were R^2^ = 0.758 and RMSE = 6.855; *p* < 10^−4^.

Carbohydrate based on PLSR = 144.181 − 769.472 X1 + 754.726 X2 + 44.451(7)

X3 was not significant at the 0.01 level. The values obtained for the model were R^2^ = 0.771 and RMSE = 6.667; *p* < 10^−4^.

**Figure 4:**
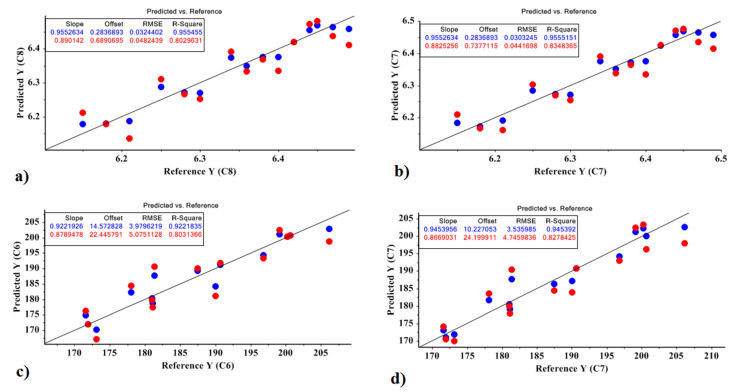
MLR prediction models for predicting the amount of carbohydrate and sugar in the Agria cultivar: (**a**) the amount of sugar based on the PCR method; (**b**) the amount of sugar based on the PLSR method; (**c**) the amount of carbohydrate based on the PCR method; (**d**) the amount of carbohydrate based on the PLSR method.

Equations (8)–(11) represent the model obtained by the MLR model for the Agria cultivar based on the optimal factors of the PCR and PLSR models for predicting the amount of sugar and carbohydrate. Correlation plots are shown in Figure 4.

Sugar based on PCR = 6.341 − 3.710 X1 − 5.996 X4 − 12.697 X6 + 0.002(8)

X2, X3, X5, and X7 were not significant at the 0.01 level, so they were deleted in the model. The values obtained for the model were R^2^ = 0.802 and RMSE = 0.048; *p* < 10^−4^.

Sugar based on PLSR = 5.723 + 3.935 X1 + 3.871 X2 + 2.224 X3 + 0.005(9)

X4, X5, and X6 were not significant at the 0.01 level. The values obtained for the model were R^2^ = 0.834 and RMSE = 0.044; *p* < 10^−4^.

Carbohydrate based on PCR = 187.293 − 357.904 X1 − 464.882 X2 −750.416 X3 + 25.756(10)

X4 and X5 were not significant at the 0.01 level. The values obtained for the model were R^2^ = 0.803 and RMSE = 5.075; *p* < 10^−4^.

Carbohydrate based on PLSR = 187.293 − 400.888 X1 − 392.236 X2 + 331.148 X3 + 851.462 X5 + 22.524(11)

X4 and X6 were not significant at the 0.01 level. The values obtained for the model were R^2^ = 0.827 and RMSE = 4.745; *p* < 10^−4^.

**Figure 5:**
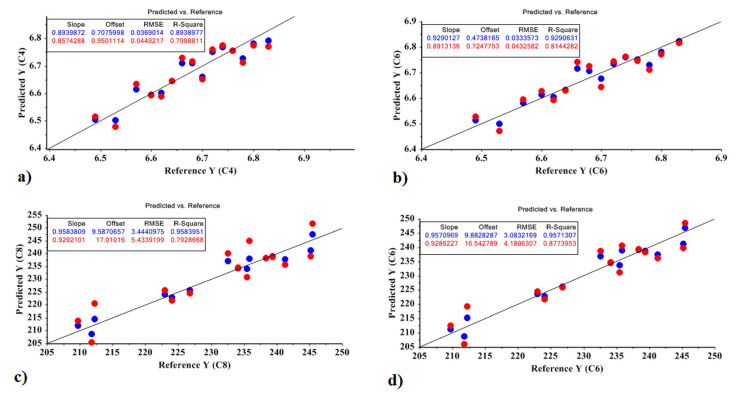
MLR prediction models for predicting the amount of carbohydrate and sugar in the Jelly cultivar; (**a**) amount of sugar based on PCR method, (**b**) amount of sugar based on PLSR method, (**c**) amount of carbohydrate based on PCR method, (**d**) amount of carbohydrate based on PLSR method.

In addition, Equations (12)–(15) represent the model obtained by the MLR method for the Jelly cultivar, based on the optimal factors of the PCR and PLSR models for predicting the amount of sugar and carbohydrate. Correlation plots are shown in Figure 5.

Sugar based on PCR = 6.674 + 6.769 X2 + 7.617 X3 + 0.002(12)

X1 was not significant at the 0.01 level, so are deleted in the model. The values obtained for the model were R^2^ = 0.799 and RMSE = 0.044; *p* < 10^−4^.

Sugar based on PLSR = 5.723 + 4.889 X1 + 4.256 X3 + 0.001(13)

X2, X4, and X5 were not significant at the 0.01 level. The values obtained for the model were R^2^ = 0.834 and RMSE = 0.044; *p* < 10^−4^.

Carbohydrate based on PCR = 230.352 − 153.711 X1 + 562.065 X2 − 726.019 X4 + 29.527(14)

X3, X5 and X7 were not significant at the 0.01 level. The values obtained for the model were R^2^ = 0.793 and RMSE = 5.433; *p* < 10^−4^.

Carbohydrate based on PLSR = 230.352 + 596.312 X1 + 199.290 X2 + 554.525 X3 + 17.477(15)

X4 and X5 were not significant at the 0.01 level. The values obtained for the model were R^2^ = 0.827 and RMSE = 4.745; *p* < 10^−4^.

**Figure 6:**
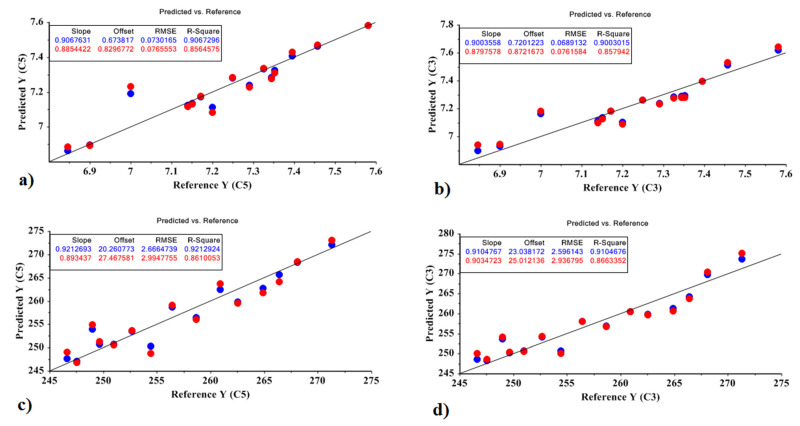
MLR prediction models for predicting the amount of carbohydrate and sugar in the Sante cultivar: (**a**) the amount of sugar based on the PCR method; (**b**) the amount of sugar based on PLSR method; (**c**) the amount of carbohydrate based on the PCR method; (**d**) the amount of carbohydrate based on the PLSR method.

Equations (16)–(19) represent the model obtained by the MLR method for the Sante cultivar, based on the optimal factors of the PCR and PLSR models for predicting the amount of sugar and carbohydrate. Correlation plots are shown in Figure 6.

Sugar based on PCR = 7.226 − 9.693 X1 + 9.917 X2 + 0.005(16)

X3 and X4 were not significant at the 0.01 level, so they were deleted in the model. The values obtained for the model were R^2^ = 0.856 and RMSE = 0.076; *p* < 10^−4^.

Sugar based on PLSR = 7.226 + 13.164 X1 + 0.005(17)

X2 was not significant at the 0.01 level. The values obtained for the model were R^2^ = 0.834 and RMSE = 0.044; *p* < 10^−4^.

Carbohydrate based on PCR = 257.342 − 297.107 X1 + 522.111 X2 + 8.968(18)

X3 and X4 were not significant at the 0.01 level. The values obtained for the model were R^2^ = 0.861 and RMSE = 2.994; *p* < 10^−4^.

Carbohydrate based on PLSR = 257.342 + 557.424 X1 + 8.624(19)

X2 was not significant at the 0.01 level. The values obtained for the model were R^2^ = 0.866 and RMSE = 2.936; *p* < 10^−4^.

**Figure 7:**
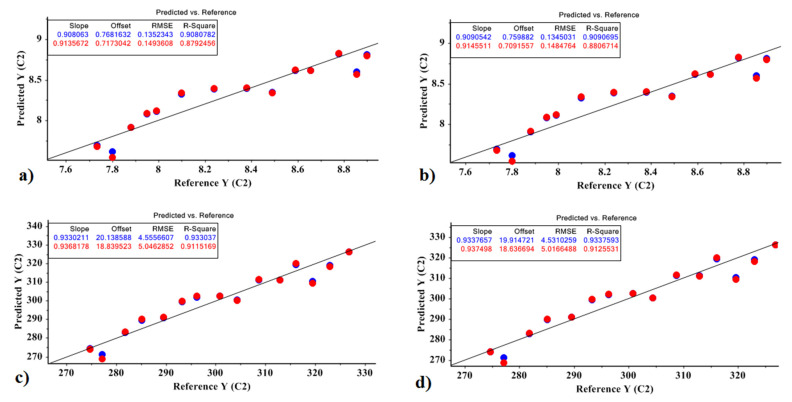
MLR prediction models for predicting the amount of carbohydrate and sugar in the Marfona cultivar: (**a**) the amount of sugar based on the PCR method; (**b**) the amount of sugar based on the PLSR method; (**c**) the amount of carbohydrate based on the PCR method; (**d**) the amount of carbohydrate based on the PLSR method.

Finally, Equations (20)–(23) represent the model obtained by the MLR method for the Marfona cultivar, based on the optimal factors of the PCR and PLSR models for predicting the amount of sugar and carbohydrate. Correlation plots are shown in Figure 7.

Sugar based on PCR = 8.355 − 30.676 + 0.022(20)

The values obtained for the model were R^2^ = 0.879 and RMSE = 0.149; *p* < 10^−4^.

Sugar based on PLSR = 8.355 − 30.701 X1 + 0.022(21)

The values obtained for the model were R^2^ = 0.880 and RMSE = 0.148; *p* < 10^−4^.

Carbohydrate based on PCR = 300.670 − 1227.29 X1 + 20.754(22)

The values obtained for the model were R^2^ = 0.911 and RMSE = 5.046; *p* < 10^−4^.

Carbohydrate based on PLSR = 300.670 − 1228 X1 + 25.166(23)

The values obtained for the model were R^2^ = 0.912 and RMSE = 5.016; *p* < 10^−4^.

### 3.5. Support Vector Machine (SVM)

To classify the samples, two methods were used—the C-SVM and the Nu-SVM—which contain four kernel functions: the radial basis function, the sigmoid function, the polynomial function, and the linear function. These methods have penalty coefficients of “C and Nu”, and a kernel coefficient of “γ”, which is used by a network search algorithm. Seventy percent of the data were used for training and 30 percent were used for testing; all input weights were one. The results of the SVM method are summarized in Table 4.

**Figure 8:**
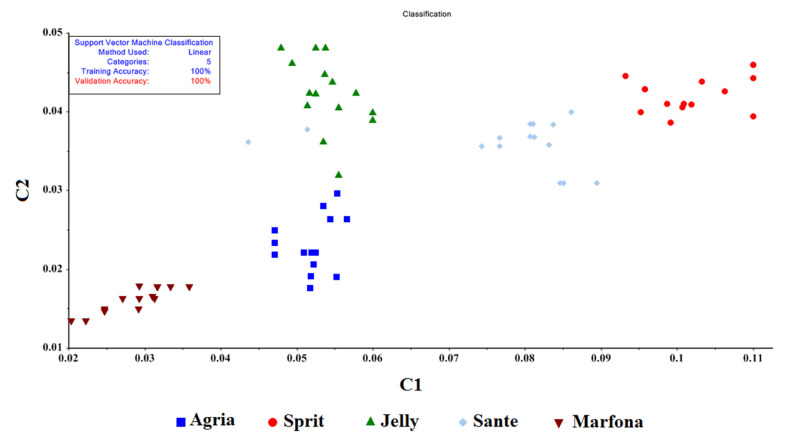
Classification of potato cultivars with the C-SVM (linear) function.

For the C-SVM method, the linear function had an accuracy of 100% in both training and validation for the five groups of potatoes. Figure 8 presents the classification accuracy results for the linear function, with 100% accuracy.

## 4. Discussion

The e-nose tested in this study provided effective discriminations among certain potato cultivars based on analyses of sample volatiles using specific statistical methods for e-nose data analysis, as indicated in subsequent discussions. Radar diagrams were used to observe the differences in patterns (fingerprints) between different potato cultivars. Such diagrams are used in various applications in the food industry (especially in the electronic nose). The gas sensors MOS, TGS813, and MQ135 showed the highest responses to the sample odors, while the sensors MQ136, MQ4, and MQ9 showed the lowest responses. Almost all the sensors showed a higher response for carbohydrates than for sugars. Knowing the response power of each sensor to different VOCs can help determine the different characteristics of the products; the most relevant and effective sensors with the maximum response to VOCs can be selected in the design of the electronic nose sensor array. The selection of the sensors with the suitable responses helps in the consideration of the transient response, to reduce the response time of the system. Moreover, knowing the role of the higher-level sensors in the data processing phase can reduce the complexity of the analysis, because sometimes additional variables in the data can lead to some problems, such as overtraining in data analysis [26]. For example, the results of research by Kiani, Minaei and Ghasemi-Varnamkhasti [11] showed that the fingerprint of mint leaves changes with drying. In addition, as in our research, they determined the efficiency of the sensors using radar diagrams and introduced the most effective and least effective ones during the drying time. In another study, Ran, et al. [27] used radar diagrams to investigate the effect of sensors on the electronic nose, and also to investigate electronic language in detecting the physicochemical properties of chicken powder. In another study, Wang, et al. [28] examined the performance of electronic nose sensors in detecting volatile compounds in pork at different cooking times/temperatures; they found that the sensors that were in SHS (superheated steam) mode performed better than other sensors. Huang, et al. [29] used an electronic nose to detect different types of volatile compounds in eels, for three modes of curing, steaming, and grilling; according to the radar diagram in their research, the fingerprints in all three modes of cooking were very similar. They also found that after cooking, the intensity of the smell of the samples intensified and the efficiency of the sensors changed.

The relationship between electronic nose signals and the predictions of amounts of sugar and carbohydrates was described by the PLSR and PCR models, and the MLR method was used to build the PLSR and PCR models. The highest accuracy of prediction of the sugar and carbohydrate parameters was related to the Marfona cultivar. The PCR and PLSR models were able to predict the amounts of sugar and carbohydrates with an accuracy of about 0.90. The lowest accuracies of prediction of the sugar and carbohydrate parameters were related to the Sprite cultivar. The PCR and PLSR models were able to predict the amounts of sugar and carbohydrates with an accuracy of about 0.80. The highest accuracy of the model in predicting the amounts of sugar and carbohydrates was related to the Marfona cultivar, with an accuracy of about 88% for predicting the amount of sugar and an accuracy of about 92% for predicting the amount of carbohydrates. In general, in all cultivars, the accuracy of predicting the amount of carbohydrates was somewhat better than the accuracy of predicting the amount of sugar. Since the sugar and carbohydrate models have good performance criteria, they can be considered reliable and acceptable models for prediction. A good correlation was found between the experimental and theoretical predictions for carbohydrates and sugars. 

Abu-Khalaf [30] studied the quality parameters of olive oil using PLS models to analyze the chemical data and the EN. The results illustrated that the EN could model the acidity parameter with good performance. The correlation coefficients obtained, using the PLS model, for the calibration and validation of acidity were 0.87 and 0.87,, respectively. Zhang, et al. [31] reported similar results using the PLSR method and an electronic nose for grapes, with R2 of 0.93. Zhou and Zeng [12] applied partial least squares spectroscopy and linear audit analysis (PLS-LDA) and found similar results, with an R^2^ of 0.96.

The results of Kiani, Minaei and Ghasemi-Varnamkhasti [11] with an electronic nose and PLS, PCR, and MLR models, used in estimating the drying time of mint leaves, were very consistent with the results of our research. With these models, they were able to estimate the drying time of mint leaves with 93% accuracy. Zaki Dizaji, et al. [32] used the electronic nose technique and the PLS, PCR, and MLR models to predict the quality of sugarcane syrup based on the purity and the percentage of refined sugar. They stated that the accuracy of the PLS method for this purpose was 77 and 71%, respectively; they also reported that the accuracy of the PCR method was 72% and 68%, respectively. They further stated that the accuracy of the PLS method for this purpose was 82% and 74%, respectively. In another study conducted by Zhou, Fan, Tan, Peng, Cai and Zhang [12] to predict the amount of linalool in Osmanthus perfume, using an electronic nose, R2 for the PCR and MLR methods was reported at 0.736 and 0.895, respectively, and the RMSE was reported at 3.98 and 10.10, respectively. Gu, et al. [33] investigated the contamination of milled rice with an electronic nose based on the PLSR and SVM models and reported accuracies of 0.913–0.877 and 0.983–0.924, respectively. Du, et al. [34] evaluated the ability of an electronic nose to identify the SSC and TA of red meat kiwi fruit using the PLSR and SVM models; their results were R2 > 0.90 and R2 = 0.99, respectively.

Xu, et al. [35] evaluated the quality of tea using an electronic nose, an electronic tongue, and electronic vision, with the PLSR method. They were able to predict the amount of amino acids with an accuracy of 0.865, the amount of catechins with an accuracy of 0.888, the amount of polyphenols with an accuracy of 0.668, and the amount of caffeine with an accuracy of 0.791. Another study conducted by Zhang, et al. [36] to evaluate flavor improvement in Antarctic krill defluoridated hydrolysate by maillard reaction, using a PLSR sensor array and model, claimed that the PLSR results showed a very high correlation (100%) between the variables, which were the sensory properties and the volatile compounds.

Zhang, et al. [37] predicted some quality indicators of kiwifruit using an electronic nose and chemometrics. According to their report, the PLSR and SVM models predicted the ripeness, SSC, and firmness of kiwifruit with an accuracy of 0.9928, 0.9143, and 0.9290, respectively. In a study conducted by Gu, et al. [38] on the rapid detection of infection levels in milled rice, using the PLSR method and the electronic nose method, rice infection levels were able to be estimated, with R2 = 0.864 and RMSE = 0.235. In another study conducted by Tian, et al. [39] on the effect of storage time and packaging methods on the freshness of dried Lycium, using chemometrics and electronic nose methods, it was concluded that the accuracy and capability of the PLS and MLR models in this field were equal to 0.9316 and 0.9330, respectively.

In other studies, similar results have been reported by some researchers. To predict the contamination of Sitophilus granarius in wheat grain by chemometrics and electronic nose methods, researchers reported that the MLR method can detect the uric acid content and protein content with (a) R2 = 0.958 and, RMSE = 1.401 and (b) R2 = 0.978 and RMSE = 0.275, respectively [40].An electronic nose was used for the early detection of Botrytis Cinerea infection in tomatoes, and it was able to detect infection in tomato plants with a PCA-based MLR model with 87.35% accuracy and an RMSE = 07.01 [41]. An electronic nose was also used to detect cheating in mutton mixed with pork. The accuracy of the results obtained with the MLR and PLS models for the electronic nose and electronic language was reported to be 91% and 98%, respectively [42]. Finally, to classify Sitophilus oryzae contamination in rice grain with the electronic nose, the MLR method reportedly estimated protein and uric acid levels of 0.972 and 0.997, respectively, and also reported RMSE of 2.08 and 1.05, respectively [43].

In the SVM model, based on a five-group classification, the classification accuracy was more than 94% for all models. In addition, in the C-SVM method with linear functions, the highest classification accuracy for learning and validation data was 100%. Previously, Karami, Rasekh, and Mirzaee-Ghaleh [7], determined the shelf life of edible oil using an electronic nose. They were able to correctly classify the oil samples with 100% accuracy using the C-SVM method. Gorji-Chakespari et al. [33] reported 99% accuracy in classifying Damascus rose essential oil by the SVM method. Karami, Rasekh, and Mirzaee-Ghaleh [8] also found 98% and 97% accuracies in training and validating SVM methods with linear kernel functions for oil oxidation detection. In a study conducted by Ghasemi-Varnamkhasti et al. [34] to describe the freshness of strawberries in polymer packaging, accuracies of 86.4% and 50.6%, respectively, were reported for training and validation using the C-SVM method, while accuracies of 85.2% and 55.6%, respectively, were reported for training and validation using the Nu- SVM and radial kernel function method. Rasekhet al. [35] classified edible essential oils into two groups and six groups using the SVM method and achieved a classification accuracy of 100% in all models for the two-group classification, i.e., the classification of essential oils of medicinal plants from fruit extracts. For the six-group classification, based on the type of extract, the polynomial and RBF functions had a classification accuracy of 98.9 in the C-SVM method, while in the Nu-SVM method the classification accuracy of linear functions and RBF was 100% in learning and 98.9% in validation. Similar results have been reported for other crops, such as grape leaves [44], fruit juices [45], essential oils [4,16], coffee bean [46], corn [47], and cucumbers [48].

## 5. Conclusions

A portable e-nose device, consisting of nine MOS gas sensors used in combination with an SVM statistical method, was used in this study to classify five potato cultivars. The classification of the potato cultivars was accomplished with 100% accuracy. These methods could possibly be used to indirectly detect other mineral and nutritional elements in agricultural products, following slightly more extensive studies to confirm the actual levels of elements in agricultural products, which could be correlated with the e-nose sensor array outputs. In addition, the PCR, PLSR, and MLR methods were used to predict the amount of sugar and carbohydrate in potatoes. The results of this study showed that the electronic nose is a suitable tool for predicting the amount of carbohydrate and sugar in potatoes, which can be generalized by these models to other cultivars as well. The carbohydrate and sugar models showed high predictive performance and exhibited the characteristics of a good model. This technique will be very useful in identifying potato varieties for factories and processing plants

## Figures and Tables

**Table 1:** Analysis of variance of chemical and mechanical parameters of potato cultivars.

Sources	Degrees of Freedom	Mean of Squares
Sugar	4	2.198 **
Error	10	0.0003
Total	14	
Carbohydrate	4	8184.567 **
Error	10	294.23
Total	14	
Cultivar (C)	4	0.002 **
Loading speed (V)	2	0.001 **
C*V	8	0.000 **
Error	90	0.000
Total	104	

** significant at *p* ≤ 0.01.

**Table 2:** Results of Duncan mean comparison testing for sugar content, carbohydrate content, and toughness of potato cultivars (α = 0.01).

	Sprit	Agria	Jelly	Sante	Marfona
Sugar	8.153 ^a^	6.183 ^d^	6.117 ^e^	7.203 ^b^	6.493 ^c^
Carbohydrate	266 ^a^	179 ^b^	237 ^a^	276.7 ^a^	159.3 ^b^
Toughness (V1)	0.103 ^e^	0.109 ^d^	0.117 ^c^	0.126 ^b^	0.135 ^a^
Toughness (V2)	0.1 ^e,f^	0.097 ^f^	0.113 ^c,d^	0.11 ^d^	0.117 ^c^
Toughness (V3)	0.099 ^e,f^	0.097 ^f^	0.113 ^c,d^	0.108 ^d^	0.117 ^c^

The letters a–f describe significant differences between the obtained means. Means with the same letters do not differ significantly. V1, V2 and V3 describe loading speeds of 10, 40, and 70 mm per minute, receptively.

**Table 3:** PLSR and PCR model analysis results for predicting sugars, carbohydrates, and toughness parameters.

Cultivars	Chemical Parametrs	Model	R^2^_cal_	R^2^_val_	RMSE_cal_	RMSE_val_	Offset_cal_	Offset_val_	Optimal Factor
Sprite	Carbohydrate	PCR	0.877	0.837	4.802	5.937	17.636	33.347	3
		PLSR	0.898	0.799	4.380	6.596	14.672	16.599	3
	Sugar	PCR	0.893	0.866	0.054	0.065	0.611	1.077	3
		PLSR	0.904	0.826	0.051	0.074	0.543	0.582	3
Agria	Carbohydrate	PCR	0.922	0.799	3.082	5.295	14.574	33.939	5
		PLSR	0.945	0.813	2.582	5.117	10.227	23.829	6
	Sugar	PCR	0.955	0.831	0.022	0.046	0.282	0.756	7
		PLSR	0955	0.820	0.022	0.047	0.282	0.834	6
Jelly	Carbohydrate	PCR	0.958	0.823	2.352	5.187	9.583	18.859	7
		PLSR	0.957	0.814	2.388	5.317	9.847	27.443	5
	Sugar	PCR	0.893	0.819	0.031	0.044	0.708	1.584	3
		PLSR	0.929	0.772	0.025	0.049	0.473	1.026	5
Sante	Carbohydrate	PCR	0.921	0.872	2.177	2.963	20.254	3.885	4
		PLSR	0.910	0.848	2.322	3.232	23.040	46.55	2
	Sugar	PCR	0.906	0.874	0.059	0.074	0.674	0.992	4
		PLSR	0.900	0.859	0.061	0.078	0.720	1.062	2
Marfona	Carbohydrate	PCR	0.933	0.923	4.241	4.859	20.133	21.208	1
		PLSR	0.933	0.923	4.218	4.859	19.916	21.046	1
	Sugar	PCR	0.908	0.895	0.125	0.143	0.768	0.781	1
		PLSR	0.909	0.895	0.125	0.143	0.759	0.775	1

**Table 4:** Results and comparison of Nu-SVM and C-SVM models subjected to the kernel functions.

Kernel Function	C-SVM ^1^				Nu-SVM ^1^			
	c	γ	Train	Validation	Nu	γ	Train	Validation
linear	0.1	1	100	100	1	0.99	93.33	98.67
Polynomial	0.01	1	94.67	94.67	0.01	0.25	97.33	98.66
Radial basis function	0.01	0.1	100	98.67	0.255	1	97.33	96.00
sigmoid	0.01	0.1	100	98.66	0.01	1	98.67	94.67

^1^ Statistical analysis models used for data analysis: Nu-SVM = Nu-penalty coefficient support vector machine; C-SVM = C-penalty coefficient support vector machine. γ = core coefficient.

## Data Availability

The datasets used and/or analyzed during the current study are available from the corresponding author on reasonable request.
